# Reliability of Multiple Mini-Interviews and traditional interviews within and between institutions: a study of five California medical schools

**DOI:** 10.1186/s12909-017-1030-0

**Published:** 2017-11-06

**Authors:** Anthony Jerant, Mark C. Henderson, Erin Griffin, Julie A. Rainwater, Theodore R. Hall, Carolyn J. Kelly, Ellena M. Peterson, David Wofsy, Peter Franks

**Affiliations:** 1 Department of Family and Community Medicine, University of California, Davis, School of Medicine, 4860 Y Street, Suite 2300, Sacramento, California, 95817 USA; 2 Office of the Vice Chancellor and Dean, University of California, Davis, School of Medicine, 4610 X Street, Suite 3101, Sacramento, California, 95817 USA; 3 Research and Evaluation Outcomes Unit, University of California, Davis, School of Medicine, 4610 X Street, Sacramento, California, 95817 USA; 4 Clinical and Translational Science Center, University of California, Davis, Health System, 2921 Stockton Boulevard, Suite 1400, Sacramento, California, 95817 USA; 5 Office of Admissions, David Geffen School of Medicine at University of California, Los Angeles, 885 Tiverton Drive Suite B27, California, Los Angeles 90095 USA; 6 Division of Medical Education, University of California, San Diego, School of Medicine, 9500 Gilman Drive, mail code 0606, La Jolla, California, 92093 USA; 7 Office of Admissions, University of California, Irvine, School of Medicine, Medical Education Building, 836 Health Sciences Road, Irvine, California, 92697-4089 USA; 8 Office of Admissions, University of California, San Francisco, School of Medicine, Box 0408, 533, Parnassus Avenue, Room U-426, San Francisco, California, 94143 USA

**Keywords:** Interview as topic, Multiple mini-interview, Reproducibility of results, School admission criteria, Schools, medical

## Abstract

**Background:**

Many medical schools use admissions Multiple Mini-Interviews (MMIs) rather than traditional interviews (TIs), partly because MMIs are thought to be more reliable. Yet prior studies examined single-school samples of candidates completing either an MMI or TI (not both). Using data from five California public medical schools, the authors examined the within- and between-school reliabilities of TIs and MMIs.

**Methods:**

The analyses included applicants interviewing at ≥1 of the five schools during 2011–2013. Three schools employed TIs (TI1, TI2, TI3) and two employed MMIs (MMI1, MMI2). Mixed linear models accounting for nesting of observations within applicants examined standardized TI and MMI scores (mean = 0, SD = 1), adjusting for applicant socio-demographics, academic metrics, year, number of interviews, and interview date.

**Results:**

A total of 4993 individuals (completing 7516 interviews [TI = 4137, MMI = 3379]) interviewed at ≥1 school; 428 (14.5%) interviewed at both MMI schools and 687 (20.2%) at more than one TI school. *Within* schools, inter-interviewer consistency was generally qualitatively lower for TI1, TI2, and TI3 (Pearson’s r 0.07, 0.13, and 0.29, and Cronbach’s α, 0.40, 0.44, and 0.61, respectively) than for MMI1 and MMI 2 (Cronbach’s α 0.68 and 0.60, respectively). *Between* schools, the adjusted intraclass correlation coefficient was 0.27 (95% CI 0.20–0.35) for TIs and 0.47 (95% CI 0.41–0.54) for MMIs.

**Conclusions:**

Within and between-school reliability was qualitatively higher for MMIs than for TIs. Nonetheless, TI reliabilities were higher than anticipated from prior literature, suggesting TIs may not need to be abandoned on reliability grounds if other factors favor their use.

## Background

Unstructured or minimally structured one-on-one traditional interviews (TIs) have long been employed in medical school admissions [1]. A number of reports have raised the concern that low inter-interviewer *reliability* (i.e., consistency) may limit the ability of TIs to distinguish applicants likely to succeed in training [2, 3]. However, findings of studies examining this issue are mixed, with wide ranges of observed consistency between interview scores (e.g., Pearson’s r correlations 0.22–0.97; generalizability [G] coefficients 0.27–0.58; kappas 0.13–0.70) [1, 2, 4–10].

Partly due to concerns about inter-interviewer reliability, many schools have replaced TIs with Multiple Mini-Interviews (MMIs), in which applicants work through a series of brief, semi-structured assessment stations, each attended by a different trained rater [3, 11]. Single-school studies examining the MMI in isolation suggest the approach yields moderate to high inter-rater reliability (range of Cronbach’s alphas reported 0.65–0.98; range of G coefficients reported 0.55–0.72), and predicts aspects of subsequent academic performance [3, 12–16].

Based on the foregoing studies, some authors have concluded that MMIs have superior inter-rater reliability as compared with TIs [2–6, 12, 17]. However, prior MMI (and TI) studies have been conducted at single institutions, each employing only one of these interview types. While valuable, such studies have relatively small samples sizes, since at any given school most applicants are not selected for an interview, reducing generalizability. Studies pooling interview data from multiple schools with partially overlapping applicant pools, each inviting a different (though again partially overlapping) subset of applicants to interview, would have larger and more representative samples. Moreover, single-school interview studies have limited utility in comparing the relative reliabilities of MMIs and TIs, due to fundamental differences in designs, analytic approaches, and time frames among studies. Importantly, no studies have concurrently tested whether inter-rater reliability is higher for MMIs than for TIs by examining a common pool of applicants completing both interview types. Furthermore, no studies have examined the *between-school* reliabilities of MMIs or TIs. As key differences in MMI (and TI) implementation exist among schools, [18] high between-school reliability of the MMI and TI cannot be assumed.

Using data from the five California Longitudinal Evaluation of Admission Practices (CA-LEAP) consortium medical schools, we examined the within- and between-school reliabilities of MMIs and TIs.

## Methods

We conducted the study activities from July 2014–April 2016. We obtained ethics approval from the institutional review boards of the participating schools via the University of California Reliance Registry (protocol #683). Because of the nature of the study, neither interviewer nor interviewee consent to participate was required.

### Study population

Participants were individuals who, during three consecutive application cycles (2011–2013), completed one or more medical school program interviews at CA-LEAP schools. The five CA-LEAP schools, all public institutions, participate in a consortium to evaluate medical school interview processes and outcomes.

### Interview processes

Two schools (MMI1 and MMI2) used MMIs, with 10 and 7 individually scored 10-min stations, respectively, generally adapted from commercially marketed content. [19] At both schools, all stations were multidimensional. Interpersonal communication ability was considered at every station, along with one or more additional competencies (e.g., integrity/ethics, professionalism, diversity/cultural awareness, teamwork, ability to handle stress, problem solving), rated using a structured rating form. At both MMI schools, stations were attended by one rater, except for a single station at MMI2 (two raters). At both schools, raters included physician and basic science faculty and alumni, medical students, and high-level administrative staff. At MMI1, raters also included nurses, patients, lawyers, and other community members. Raters at both schools received 60 min of training before each application cycle; MMI2 raters also received a 30-min re-orientation prior to each MMI circuit. The raters were not given any information about applicants. They interacted directly with applicants at some stations, and observed applicant interactions (e.g., with actors) at others. Raters at both schools assigned a single global performance score (with higher scores indicating better performance), though the scales employed differed between schools (0–3 points at MMI1, 1–7 points at MMI2).

Three schools (TI1, TI2, and TI3) used TIs. At each school, applicants completed two 30–60 min unstructured interviews, one with a faculty member and one with a medical student or faculty member. All interviewers received 60 min of training before each application cycle. At TI1 and TI2, interviewers reviewed the candidate’s application prior to the interview, although academic metrics were redacted at school TI1. TI3 interviewers reviewed the candidate’s application only after submitting their interview ratings. All interviewers rated applicants on standardized scales, though the rating approaches and scales employed differed among schools. At both schools TI1 and TI3, interviewers assigned a single global interview rating, though the scales employed differed (exceptional, above average, average, below average, unacceptable at TI1; unreserved enthusiasm, moderate enthusiasm, or substantial reservations at TI3). At school TI2, interviewers rated candidates on a 1–5 point scale in four separate domains (thinking/knowledge, communication/behavior, energy/initiative, and empathy/compassion), and the domain scores were then summed to yield a total interview score (range 4–20).

### Measures

The total interview scores were the means of individual station (MMI) or interview (TI) scores, converted to z-scores (mean = 0, standard deviation = 1) based on all scores within a given school and year. Applicant characteristics included age; sex; race/ethnicity category; self-designated disadvantaged (DA) status (yes/no); cumulative grade point average (GPA); and total Medical College Admissions Test (MCAT) score.

### Analyses

Analyses were conducted using Stata (version 14.2, StataCorp, College Station, TX). For the 2012 and 2013 application cycles, the analyses include data from all five schools. For 2011, TI3 provided no data. We first conducted analyses of inter-interviewer (for TIs) or inter-rater (for MMIs) reliability *within* each institution. For each of the two MMI schools, we examined the internal consistencies of MMI station scores with Cronbach’s α. For each of the three TI schools, we examined both the correlations of TI scores with Pearson’s r, and the internal consistencies of TI scores with Cronbach’s α (the latter reported to facilitate comparisons with the two MMI schools) [20, 21].

Next, we examined the pairwise Pearson correlations among interview scores obtained by applicants who interviewed at more than one school, TI and/or MMI.

Finally, we conducted analyses examining the intraclass correlation coefficients (ICCs) observed between MMI schools and among TI schools. All applicants who interviewed at one or more TI school contributed to the TI ICC analyses, and all applicants who interviewed at one or more MMI school contributed to the MMI ICC analyses. For both MMI and TI analyses, we developed mixed linear models [22] with applicants as random effects to derive the ICCs for interview z-scores at TI and MMI schools Both the TI and MMI analyses were conducted with and without adjusting for the following (potentially confounding) fixed effects: applicant characteristics (socio-demographics, DA status, and metrics), number of interviews, number of prior interviews, interview date within interview season, and interview year. In each case the ICC of interest (ICC [1]) was the ratio of the variance component associated with the random effect (applicant) divided by the total variance [23]. The use of mixed models allowed adjustment for the nesting of observations (applicant interviews) within applicants, for those with more than one interview. Simultaneously, the analysis allowed examination of the consistency of performance among the three TI schools and between the two MMI schools (the ICCs).

## Results

There were 4993 individuals with at least one interview at a CA-LEAP school during the study period; their socio-demographics and academic metrics are shown in Table 1 (next page). Of these, 3226 (65%), 1180 (24%), 439 (8.8%), 127 (2.5%), and 21 (0.4%) interviewed at one, two, three, four, or all five schools, respectively; 428 (14.5%) interviewed at both MMI schools; 687 (20.2%) interviewed at more than one TI school; and 119 (2.4%) interviewed in more than one year.

**Table 1:** Socio-demographics and academic metrics of interviewees at CA-LEAP schools, 2011–2013

Characteristic	Interviewees (*N* = 4993)^a^
Age category, number (%)	
18 to <23	1646 (33.0)
23	1172 (23.5)
24	778 (15.6)
≥ 25	1397 (28.0)
Female, number (%)^b^	2378 (47.6)
Race/Ethnicity category, number (%)	
Non-Hispanic Black	276 (5.5)
Non-Hispanic Asian	1775 (35.5)
Non-Hispanic White	1774 (35.5)
Non-Hispanic Other	556 (11.1)
Hispanic (any race)	612 (12.3)
Self-designated disadvantaged, number (%)	958 (19.2)
Cumulative grade point average, mean (SD)^b^	3.72 (0.22)
Medical College Admissions Test total score, mean (SD)	33.6 (3.7)
Admission cycle, number (%)	
2011	1509 (30.2)
2012	1757 (35.2)
2013	1727 (34.6)

^a^TI3 provided no data for the 2011 admission cycle

^b^Four applicants had missing gender or grade point average information

The 4993 distinct individuals in the study completed a total of 7516 interviews (4137 TIs and 3379 MMIs); Table 2 shows socio-demographics and academic metrics by interview type. As compared with individuals completing TIs, those completing MMIs were statistically significantly more likely to be from a racial/ethnic minority group, self-designate as disadvantaged, and have lower a cumulative GPA and total MCAT score.

**Table 2:** Socio-demographics and academic metrics by interview type at CA-LEAP schools, 2011-2013^a^

Characteristic	TI (*N* = 4137)^b^	MMI^c^ (*N* = 3379)	*P* value^e^
Age category, number (%)			.24^e^
18 to <23	1364 (33.0)	1152 (34.1)	
23	1039 (25.1)	781 (23.1)	
24	640 (15.5)	528 (15.6)	
≥ 25	1094 (26.4)	918 (27.2)	
Female, number (%)^d^	2033 (49.1)	1626 (48.1)	.38^f^
Race/Ethnicity category, number (%)			<.001^f^
Non-Hispanic Black	212 (5.1)	214 (6.3)	
Non-Hispanic Asian	1429 (34.5)	1253 (37.1)	
Non-Hispanic White	1529 (37.0)	1099 (32.5)	
Non-Hispanic Other	433 (10.5)	371 (11.0)	
Hispanic (any race)	534 (12.9)	442 (13.1)	
Self-designated disadvantaged, number (%)	608 (14.7)	892 (26.4)	<.001^f^
Cumulative grade point average, mean (SD)^d^	3.75 (0.21)	3.72 (0.23)	<.001^g^
Medical College Admissions Test total score, mean (SD)	34.0 (3.4)	33.5 (4.0)	<.001^g^
Admission cycle, number (%)			
2011	1061 (25.6)	1124 (33.3)	<.001^f^
2012	1506 (36.4)	1186 (35.1)	
2013	1570 (38.0)	1069 (31.6)	

^a^4993 completed a total of 7516 interviews, 4137 TIs and 3379 MMIs

^b^Three CA-LEAP schools employed Tis (TI1, TI2, and TI3); TI3 provided no data for 2011 admission cycle

^c^Two CA-LEAP schools employed MMIs (MMI1 and MMI2)

^d^Four applicants had missing gender or GPA information

^e^ *P* value for difference in characteristic between applicants completing TIs versus MMIs

^f^Chi-square test

^g^t-test

*Within* schools, correlations between interviewer ratings generally were qualitatively lower for TI1 (r 0.07, α 0.13), TI2 (r 0.29, α 0.40), and TI3 (r 0.44, α 0.61) than for MMI1 and MMI2 (α 0.68 and 0.60, respectively). *Between* school z-score correlations varied considerably (r range 0.18–0.48), with the highest correlation observed between MMI1 and MMI2 (Table 3, next page).

**Table 3:** Between-school Pearson’s r correlations of TI and MMI Z-Scores at CA-LEAP schools, 2011-2013^a,b^

	TI1	TI2	TI3	MMI1	MMI2
TI1	1.00	–	–	–	–
TI2	0.28	1.00	–	–	–
TI3	0.24	0.28	1.00	–	–
MMI1	0.18	0.36	0.23	1.00	–
MMI2	0.19	0.36	0.21	0.48	1.00

^a^N = 3379

^b^TI school 3 provided no data for the 2011 admission cycle

In an unadjusted analysis, the ICC was higher for MMI schools (0.47, 95% CI 0.40–0.54) than for TI schools (0.30, 95% CI 0.24–0.37). After adjustment for applicant characteristics, application year, and number and temporal sequencing of interview, the ICCs were similar to the unadjusted values, though qualitatively lower for TI schools: 0.27 (95% CI 0.20–0.35) for TI schools and 0.47 (95% CI 0.41–0.54) for MMI schools.

## Discussion

To our knowledge, the current study was the first to concurrently examine the within- and between-school reliabilities of unstructured TIs and of MMIs in a common pool of applicants to multiple medical schools. As such, our findings expand substantively on those of prior studies of admissions interviews, all conducted at single schools, which had smaller and less representative samples and examined only the within-school (but not the between-school) reliabilities of TIs or MMIs (but not both).

We generally found qualitatively higher within-school and between-school reliabilities for MMIs than for TIs. This is reassuring, since one goal of the MMI approach is to increase the reliability of the medical school interview process, and, potentially, predictive validity [3]. Similar ICCs were observed using unadjusted and adjusted mixed models for both MMIs and TIs, indicating little influence of applicant socio-demographics and metrics, prior interview experience, or interview timing on the reliability of either interview approach. The adjusted analyses were important to conduct given statistically significant differences in socio-demographics and academic metrics between MMI and TI participants (Table 2), likely reflecting differing missions and priorities across CA-LEAP schools.

We observed qualitatively lower internal consistency for MMI2 (α 0.60) than for MMI1 (α 0.68). Prior single-school studies have found that increasing the number of MMI stations tends to enhance reliability [12, 24, 25]. Thus, this finding likely reflects the use of only seven stations at MMI2 versus ten at MMI1, and underscores the need for schools adopting an MMI to carefully consider this design choice.

Despite the qualitatively superior between-school reliability of the MMI in our study, the between-school TI reliabilities were better than we had anticipated based on prevailing views [2–6, 12, 17]. These findings suggest that the low inter-interviewer reliability observed for TIs in some (but not all) prior single-school studies may reflect school-specific differences (e.g., interviewer training, degree of process standardization), rather than limitations inherent to the TI approach. In particular, the qualitatively lower between-school reliability for the TI may reflect intentional differences between schools with respect to their goals, a distinction that might be easier to achieve with unstructured TIs as compared with the more standardized MMI approach. Therefore, abandoning TIs on the grounds of qualitatively lower reliability may not necessarily be advisable. This may be particularly true since limited research suggests that the reliability of traditional interviews (within and between schools) might be improved through relatively minor process enhancements. These may include, but are not necessarily limited to, increased standardization of interview questions, and greater efforts to calibrate interviewers (e.g., by providing sample answers for evaluating applicant responses and, within schools, affording opportunity for discussion among interviewers) [1, 26]. Nonetheless, we emphasize that the foregoing comments are speculative, best viewed as hypotheses to be further tested in multi-school studies.

A key strength of our multi-institutional study was the large sample of applicants to five public medical schools in California (one of the most socio-demographically diverse states). Our study also had some limitations. The extent to which the findings may apply to non-CA-LEAP schools is uncertain. From a strict measurement perspective, our assessments of reliability were not pure, since each interview (two at each TI school, 10 stations at MMI1, and 7 stations at MMI2) was conducted by an independent rater assessing an independent encounter. We focused on the within- and between school reliabilities of TIs and MMIs and did not address how differences in TI and MMI reliability may affect their predictive validity – in other words, their association with future clinical rotation performance, licensing examination scores, and other relevant outcomes. It is anticipated that future CA-LEAP studies will address this important issue. As others have also observed, [12] current evidence for the predictive validity of the MMI stems from single-medical school studies (all conducted outside of the U.S.). Such studies are limited by the lack of concurrent examination of TI validity, and by the relatively small proportion of interviewees who matriculate at any given school. By comparison, in a multi-school consortium pool of interviewees, a relatively higher proportion would be anticipated to matriculate at one of the schools, permitting a more robust examination of MMI predictive validity and concurrent comparison with TI predictive validity.

## Conclusions

In conclusion, in analyses of data from a common pool of applicants to five California medical schools, we found qualitatively higher within- and between-school reliabilities for MMIs than for TIs. Nonetheless, the within- and between-school reliabilities of TIs were generally higher than anticipated based on prior literature, suggesting that perhaps TIs need not be abandoned for the sake of reliability concerns, especially if other factors favor their use at a particular institution.

## Abbreviations

CA-LEAP: California Longitudinal Evaluation of Admission Practices
DA: disadvantaged
GPA: grade point average
ICC: intraclass correlation coefficient
MCAT: Medical College Admissions Test
MMI: Multiple Mini-Interview
MMI1 and MMI2: study schools using an MMI
SD: standard deviation
TI: traditional interview
TI1, TI2, and TI3: study schools using TIs
